# Influence of Silica Exposure for Lung Silicosis Rat

**DOI:** 10.1155/2021/6268091

**Published:** 2021-12-13

**Authors:** Jie Jiao, Li Li, Wu Yao, Weidong Qin, Changfu Hao, Lingeng Lu

**Affiliations:** ^1^Henan Provincial Institute for Occupational Health, Zhengzhou, Henan, China; ^2^The First Affiliated Hospital of Zhengzhou University, Zhengzhou, Henan, China; ^3^School of Public Health, Zhengzhou University, Zhengzhou, Henan, China; ^4^Yale School of Public Health, Yale University, New Haven, Connecticut 06510, USA

## Abstract

**Objective:**

To investigate the influence of silica exposure on the expression of connective tissue growth factor (CTGF), transforming growth factor beta-1 (TGF-*β*1), and platelet-derived growth factor (PDGF) in lung silicosis rat.

**Methods:**

Wistar rats were divided into an experimental group and a control group. In the experimental group, rats were exposed to silica by intratracheal instillation. In the control group, rats were exposed to physiological saline by intratracheal instillation. After 45 days, we compared the level of fibrosis and CTGF, TGF-*β*1, and PDGF in the lungs by immunohistochemistry or reverse transcription-polymerase chain reaction between the two groups.

**Results:**

The results showed that the expression levels of CTGF, TGF-*β*1, and PDGF mRNA were significantly higher in the experimental group than those in the control group (*P* < 0.05). The positive staining of CTGF, TGF-*β*1, and PDGF mRNA was found in the cytoplasm, especially in the silicotic nodules of the hyalinisation section and cell endochylema of the alveolar macrophages, type II pneumonocytes, and lung tracheal epithelium. There were significantly positive correlations between CTGF, TGF-*β*1, and PDGF expressions (*P* < 0.05). A protein–protein interaction analysis showed interactions between TGF-*β*1, CTGF, and PDGF.

**Conclusions:**

TGF-*β*/CTGF signaling pathway plays an important role in silicosis. Silicon dioxide exposure can induce the expression of CTGF, TGF-*β*1, and PDGF.

## 1. Introduction

Chronic silicosis is a common occupational disease caused by long-term inhaled silica dust at low levels in a dust-producing workplace. It is different from “accelerated silicosis,” in which the latency period is 5-10 years, and the “acute silicosis” which was caused by the exposure to high concentrations of silica for short periods [1]. Its pathological characteristics include lung tissue fibrosis, alveolar epithelium trauma, and increased fibroblast proliferation and collagen protein. Although the incidence of new silicosis diagnoses in the world shows a downward trend, the incidence is still high in some countries and it has irreversible damage; the prevention of silicosis is of utmost importance.

Recently, the molecular mechanisms of silicosis due to silica exposure have attracted more attention. Inflammation-mediated lung fibrosis is one principal hypothesis. The key point of this hypothesis is that inflammatory networks are activated when inhaled silica particles in the lungs are engulfed by macrophages, resulting in the release of inflammatory cytokines into the alveolar space [2]. Consequently, silicosis occurs due to these chronic inflammatory stimuli. Previous studies have suggested that several cellular cytokines play an important role in the development of fibrosis in different organs, which include connective tissue growth factor (CTGF), transforming growth factor-beta1 (TGF-*β*1) and platelet-derived growth factor (PDGF) [3–5]. CTGF, a member of the CCN gene family (CTGF/CCN2), is a critical profibrogenic cytokine in various fibrotic disorders [6–10]. It has been shown that CTGF can promote mitosis, induction of cellular proliferation, and synthesis of extracellular matrix (extracellular matrix, ECM) and participate in the repair processes of tissue injury [11–14]. CTGF is often coexpressed with TGF-*β*1 in various fibrotic disorders [15] and acts as a potent downstream mediator of TGF-*β*1, orchestrating with each other in the pathogenesis of fibrosis [16]. As a profibrogenic cytokine, TGF-*β*1 increases synthesis of and stabilises extracellular matrix proteins, causing a gradual destruction of normal tissue structure and function [17].

PDGF was first identified as a serum cytokine in the 1970s, which stimulates proliferation of smooth muscle cells [18]. Overexpression of PDGF can lead to several human health disorders, including atherosclerosis, balloon injury-induced restenosis, pulmonary hypertension, organ fibrosis, and tumorigenesis [19]. Current researches showed that PDGF cooperates with TGF-*β*1, encourages the development of organ fibrosis, and is associated with pulmonary fibrosis [20, 21].

Although inflammatory cytokines such as TGF-*β*1 have been suggested to be involved in silicosis in epidemiologic studies [22, 23], the roles of the above three cytokines in silica-induced silicosis remain unknown. Therefore, we conducted this study to investigate the influence of silica exposure on the expression of CTGF, TGF-*β*1, and PDGF in lung silicosis rat.

## 2. Material and Methods

### 2.1. Experimental Animals

Forty healthy adult Wistar rats, half male and half female, weighing between 185 g and 212 g, were obtained from the Experimental Animal Centre of Henan province, whose certificate number is 410101. The living conditions of the experimental animals were a temperature of 21–25°C and a relative humidity of 50%–70%. After one week of adaptive feeding, the rats were divided randomly into the experimental and control groups. The research council's guidelines from the Henan Provincial Institute of Occupational on the care and use of laboratory animals were followed.

### 2.2. Methods

In the experimental group, the rats were exposed to instillation of silica intratracheally through a laryngoscope. A total volume of 1 ml (100 mg) of silica suspension was injected into the bronchi, immediately followed by the injection of 2 ml of air. To make the dust scatter in the whole lung, pull out the endotracheal intubation and gently pat the chest walls on both sides of the rat with the palm to disperse the dust to the whole lung and relieve the asphyxia of the rat as soon as possible. After gas was pumped back, 1 ml silica dust suspension was slowly injected into the trachea (silica dust suspension was 50 mg/ml, prepared with 0.9% NaCl solution; penicillin sodium 8000 U/1 ml was added before use to prevent lung infection; and it should be well shaken before each suction of silica dust suspension). Then, the animal is immediately upright and rotated so that the fluid is evenly distributed in the left and right lungs, the subcutaneous tissue and muscles are reset, and the skin is sutured and disinfected. In the control group, rats were exposed to physiological saline by intratracheal instillation. After 45 days, the rats were sacrificed and lung tissues were taken and washed with a physiological saline solution. Most of the lung tissues were preserved in liquid nitrogen, and a small portion was fixed by 10% neutral formalin.

### 2.3. Pathological Grading

The formalin-fixed paraffin-embedded lung tissues were stained by HE and Masson, respectively, using routine methods. HE staining can help observe the inflammatory cell infiltration, lung tissue structure, and fibrosis of lung tissue [24]. Masson staining method help see the blue collagen fibers surrounding the red hepatocyte mass; then, the color contrast can clearly distinguish the degree of fiber hyperplasia and is more conducive to the staging of lung fibrosis [25]. The pathological changes were observed using an optical microscope. Pathology grading of the silicotic nodules was conducted by referring to the *five-grade taxonomy of King* [26], which is as follows: grade 1: cell ingredients constituted of silicotic nodules and their periphery consisted of manipulus fibroblasts; grade 2: cell ingredients constituted of silicotic nodules, and their periphery consisted of major fibroblasts; collagen was not essential; grade 3: silicotic nodules constituted of mainly collagenous fibers; cell ingredients still remained; grade 4: silicotic nodules constituted of mainly collagenous fibers, cell ingredients seldom; grade 5: Silicon nodules have collagen fibrosis and fusion, with little or no cellular components, and there were very few or no fused collagenous fibers.

### 2.4. Detection of CTGF, TGF-*β*1, and PDGF

Total RNA was extracted from the fresh lung tissues of the rats using a total RNA extraction kit. The RNA quantity and quality were evalued using 1% agrose gel electrophoresis and the absorbance of RNA, respectively. The total RNA was transcribed to cDNA using AMV reverse transcriptase (Shanghai Sangon Bioengineering Technical Service Company). The mRNA levels of CTGF, TGF-*β*1, and PDGF were analyzed using RT-PCR, and *β*-actin was used as the internal reference based on the indications of the kit. PCR was performed under the following conditions: 94°C for five minutes, followed by 32 cycles of 94°C for one minute, annealing temperature for one minute, and 72°C for one minute. The primer sequences and the annealing temperature are listed in Table 1. The PCR products were separated by agarose gel electrophoresis and observed under a UV imaging system (Syngene GeneGenius, USA). The proteins of the factors were also analyzed using an immunohistochemistry method. The Biosens Digital Imaging System was used to analyze the half-quantitative analysis of the immunohistochemical results.

### 2.5. Protein-Protein Interaction Prediction

The interactions between TGF-*β*1, CTGF, and PDGF were predicted by a structure-based protein-protein interaction algorithm PrePPI [27] and STRING [28]. The parameter of the score was 0.70, and the remaining parameters were set as defaults in the STRING analysis (http://string-db.org). Two subunits of PDGF, PDGF-alpha, and PDGF-beta were used in the analyses.

### 2.6. Statistical Analyses

We used the software program SPSS 11.0 to conduct the statistical analysis. The continuous variables of normal distribution were expressed as mean ± standard deviation, the continuous variables of nonnormal distribution were expressed as median (interquartile range (IQR)), and the categorical variables were expressed as frequency (percentage (%)). For two comparisons, each value was compared by *t*-test when each datum conformed to normal distribution, while the nonnormally distributed continuous data were compared using nonparametric tests. The counting data were tested by the chi-square test. Correlation between the three cytokines was performed using the Pearson correlation; all *P* values presented are 2-sided, and a *P* value of less than 0.05 was considered significant.

## 3. Results

### 3.1. Silicosis Pathological Features

After 45 days of treatment, pathological foci of silicosis were observed in the lungs of all 20 rats instilled with dust in the experimental group; the lung volume of the silicosis group rats doubled, and the tissue texture was stiffer; oyster white needlepoint-like punctiform foci of infection were observed and felt stiffer when it was cut, and nodule felt like sand. HE staining results demonstrated that the normal lung structure was destroyed with increased cell proliferation and infiltration, a transparent silicotic nodule, thickened interstitial substance, and fibroplasias. Pathology grading of the silicotic nodule was classified by referring to the five-grade taxonomy of King; all pathological changes of the silicotic nodules were from grade one or grade two. However, no fibrosis foci were observed in the control group. The bilateral lungs of the rats appeared pink with a soft texture in the control group; HE staining showed that lung tissues of the control group had a clear structure and a thin linear alveolar wall and had no inflammatory cell infiltration in the alveoli.

### 3.2. The mRNA Level of CTGF, TGF-*β*1, and PDGF in Lung Tissues

The target genes and internal controls were amplified using RT-PCR. The levels of CTGF, TGF-*β*1, and PDGF mRNA in the experimental group were all significantly higher than those in the controls (*P* < 0.05) (Table 2). There were significantly positive correlations between mRNA expressions of CTGF, TGF-*β*1, and PDGF in the experimental group (*P* < 0.05) (Table 3); similarly, positive correlations between mRNA expressions of CTGF, TGF-*β*1, and PDGF were also seen in the control group (*P* < 0.05) (Table 3). The total correlativity between the mRNA expressions of CTGF and TGF-*β*1 in the control group was significant (coefficient correlation *R* = 0.634, *P* = 0.003); similarly, that of TGF-*β*1 and PDGF in the control group was also significant (coefficient correlation R = 0.679, *P* = 0.001) (Table 3).

### 3.3. The Protein Expression Level of CTGF, TGF-*β*1, and PDGF

**Figure 1:**
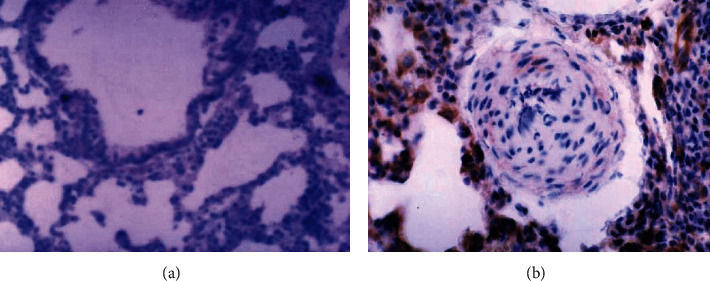
The results of immunohistochemistry of CTGF: (a) the lung tissues of the control group rats (40x); (b) the silicotic nodule of the lung tissues in the experimental group rats (40x).

**Figure 2:**
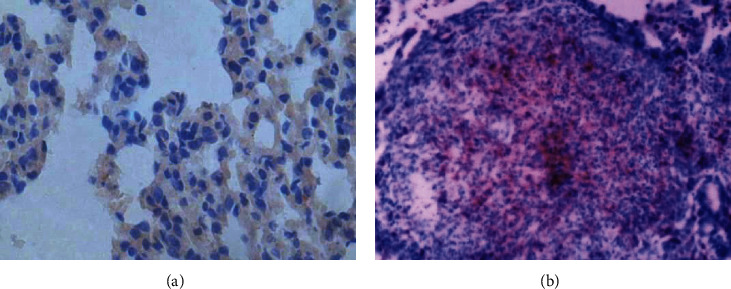
The results of immunohistochemistry of TGF-*β*1: (a) the lung tissues of the control group rats (40x); (b) the silicotic nodule of the lung tissues in the experimental group rats (40x).

**Figure 3:**
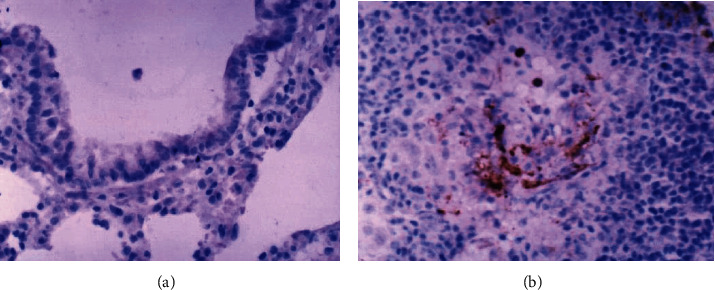
The results of immunohistochemistry of PDGF: (a) the lung tissues of the control group rats (40x); (b) the silicotic nodule of the lung tissues in the experimental group rats (40x).

**Figure 4:**
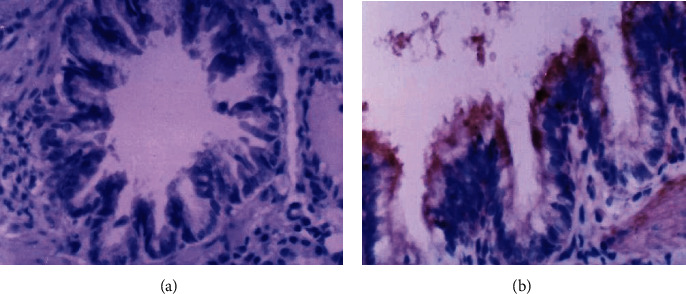
The results of positive staining and negative control of bronchiole epithelial cell immunohistochemistry: (a) the results of negative control of bronchiole epithelial cells (40x); (b) the results of positive staining of bronchiole epithelial cells (40x).

Immunohistochemical staining results showed that CTGF, TGF-*β*1, and PDGF proteins were mainly expressed in the cytoplasm, especially in the silicon nodule hyalinisation section and the cell endochylema of alveolar macrophage, type II pneumonocyte, and lung tracheal epithelium (Figures 1–4); the proteins of CTGF, TGF-*β*1, and PDGF expressed in the experimental group were higher than those in the control group (*P* < 0.05) (Table 4).

### 3.4. Protein-Protein Interactions

**Figure 5:**
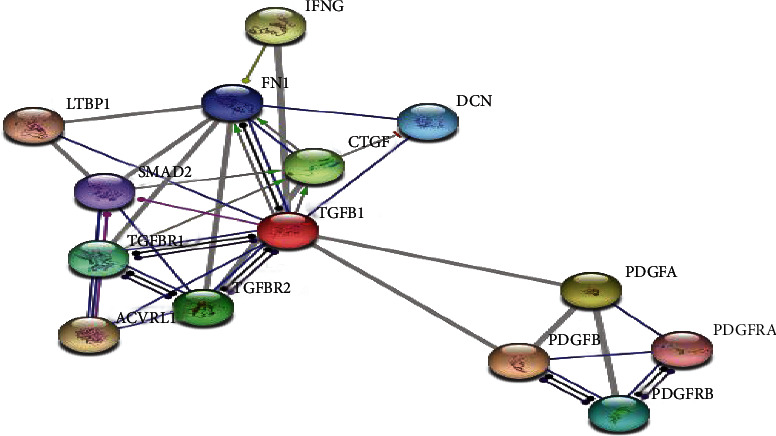
The proteins of CTGF, TGF-*β*1, and PDGF interactions using the algorithm STRING.

Xie et al. [29] evaluated the clinical relevance of TGF-*β*1 and CTGF in the diagnosis of pulmonary interstitial fibrosis in acute respiratory distress syndrome (ARDS). It was found that the mechanical work of ARDS patients was positively correlated with serum TGF-*β*1 and CTGF, and the correlation coefficients were 0.424 and 0.581, respectively. Coexpression of CTGF, TGF-*β*1, and PDGF in both experimental and control groups suggests that interactions may exist between them. Structure-based protein-protein interaction predicts that TGF-*β*1 interacts with both CTGF and PDGF-alpha with a probability of 1.0 while interacting with PDGF-beta with a probability of 0.54, and CTGF interacts with PDGF-beta and PDGF-alpha with the probabilities of 0.79 and 0.51, respectively. Similarly, direct and indirect interactions between TGF-b1 and CTGF were found using the STRING algorithm (Figure 5). Holmes et al. found that TGF-*β* stimulated cells to produce CTGF. They found a response element that can be recognized by TGF-*β* downstream factors Smad3 and Smad4 at the 168-175 base sequence of CTGF promoter. Meanwhile, the STRING algorithm found that CTGF was directly regulated by TGF-*β*1 and indirectly mediated by SMAD2, a downstream molecule of TGF-*β* pathway [30]. There was an interaction between PDGF and TGF-*β*1; however, no direct interactions were found between PDGF and CTGF in the STRING analysis.

## 4. Discussion

In this study, the effect of silica on the expression of cytokines was demonstrated, which is thought to mediate the development of silicosis in the lung. Silicon nodules formed in the rat lungs with significant pathological alterations after a 45-day exposure to silica; in contrast, no fibrosis foci were observed in the control group. There are many cytokines involved in the occurrence and development of pulmonary fibrosis, such as TGF, CTGF, PDGF, VEGF, and IGF. These cytokines and inflammatory mediators affect the inflammatory response and stimulate the formation of pulmonary fibrosis by transforming fibroblasts to proliferate and synthesize ECM. There have been a lot of studies on the role of cytokines in the formation mechanism of pulmonary interstitial fibrosis, and most of them are still cultured in vitro. In this study, it was confirmed in vivo that PDGF or CTGF, as a downstream effector of TGF or in cooperation with TGF, may play a role in the pathogenesis of silicosis. The levels of CTGF, TGF-*β*1, and PDGF mRNA and proteins in the experimental group were all significantly higher than those in the controls (*P* < 0.05). These results suggest that silica exposure could stimulate the expression of these cytokines and that CTGF, TGF-*β*1, and PDGF might be involved in the pathogenesis of silicosis. The present experimental study extends the evidence that cytokines and their interactions play an important role in the development of lung fibrosis [31–33].

CTGF promotes the synthesis of ECM, such as collagen I, collagen III, and fibronectin. Studies have revealed that CTGF was overexpressed in the process of wound healing and fibrosis of the kidneys, liver, heart, and lungs [15, 21, 34, 35]. Under the physiological state, secreted CTGF is undetectable in vivo, while under pathologic conditions, CTGF is overexpressed, stimulating cell proliferation to develop fibrotic diseases [36]. It has been reported that CTGF is an important factor mediating the pathogenesis of pulmonary fibrosis; the dificiency of CTGF may be lead to fibrosis resistance, while a CTGF-rich microenvironment makes mice susceptible to fibrosis [37]. In vitro experiments demonstrate that the expression of CTGF was upregulated in the fibrosis course [38]. CTGF is also reported to be involved in TGF-*β*1 biological activity, such as TGF-*β*1 binding to CTGF which has been shown to regulate cell proliferation and extracellular matrix components synthesis [39]. TGF-*β*1 can also induce the expression of CTGF mRNA in human skin fibroblasts. However, the mechanism of TGF-*β*/CTGF signaling pathway is still unclear. TGF-*β*/CTGF signaling pathway is one of the important pathways of signal transduction in human cells, which plays an important role in the fibrosis of various tissues and organs of the human body. There are two main transduction pathways between TGF-*β*/CTGF signaling pathways: (1) TGF-*β*/Smads/CTGF signaling pathway. This pathway mainly activates Smad-dependent and Smad-independent pathways through TGF-*β*1 for signal transduction, and then, Smad protein transduces signal into the nucleus, activates the initiation gene of CTGF, transcribes into mRNA in the nucleus, synthesizes CTGF-related proteins in the cytoplasm, and further transduces fibrosis-related signals. Aromatase inhibitors play an antipulmonary fibrosis role by inhibiting TGF-*β*1/Smad signaling pathway by affecting Smad2 and Smad3 phosphorylation [40]; (2) TGF-B/MAPK/CTGF signaling pathway, including ERK1, ERK2, p38, and c-Jun 4 pathways. Ou et al. found that TGF-*β* induces CTGF expression through ERK/ADAM17/RSK1/C/EBP*β* signaling pathway. In addition, ADAM17 and CTGF were involved in TGF-*β*-induced FN expression in human lung epithelial cells.

Studies have shown that TGF-*β*1 expression increases in the progression of lung fibrosis and could induce inflammatory cell and fibroblast to secrete interleukin-1, tumour necrosis factor-*α*, and PDGF, as well as regulate itself through autocrine mechanisms [41, 42]. With the stimulation of silica, macrophage cells produce TGF-b1 dependent on Src-ERK/AP1 pathways. This activation, however, can be inhibited by Src and ERK [43]. Previous studies [42, 44–47] have shown that pulmonary fibrosis is associated with TGF-*β*1. Wang et al. [48] reported that the inhibition of TGF-b1 could suppress silica-induced lung fibrosis in rats.

Gulumian et al. [49] have suggested that PDGF could be a serum biomarker in the early diagnosis of silicosis induced by silica and smut. *In vivo* and *in vitro* studies have demonstrated that inhaled smut could stimulate macrophages to secrete cytokines of TGF-*β*1, PDGF, and IGF [50, 51]. Perkins et al. [52] recently reported that PDGF significantly increases in human lung epithelial cells exposed to silica particles in vitro. Wu et al. [53] reported that PDGF could promote lung fibroblast proliferation only in vitro, but not *in vivo*. In contrast, other studies have demonstrated that TGF-*β*1 and PDGF play a critical role in the occurrence of pulmonary fibrosis [54, 55].

In the present study, there were significantly positive correlations between mRNA expressions of CTGF, TGF-*β*1, and PDGF in the experimental group (*P* < 0.05) (Table 3); similarly, positive correlations between mRNA expressions of CTGF, TGF-*β*1, and PDGF were also seen in the control group (*P* < 0.05) (Table 3). These findings suggest that these cytokines are coexpressed in the lungs. Increased levels of these cytokines in the experimental group also suggest that these factors may work together in the pathogenesis of silicosis. These results were consistent with previous reports [24, 38, 49, 50, 56]. Previous studies suggest that CTGF may interplay with TGF-*β*1 in the process of fibroblast proliferation and ECM production [52, 53]. PDGF and TGF-*β*1 are closely associated with fiber proliferation and deposition of ECM as well as blood vessel remodelling and regeneration of epithelial cells [57]. TGF-*β*1 could increase the expressions of PDGF and PDGFR simultaneously through unknown transcription mechanisms [12]. Moreover, Sun et al. [14] have demonstrated that Baicalein alleviated TGF-*β*1-induced type I collagen production in lung fibroblasts via downregulation of connective tissue growth factor. The protein-protein interaction theoretical analyses are consistent with findings in previous reports [12, 14, 56–58]. However, the role of these cytokines in silicosis needs further investigation through experimental interventions.

This study had several strengths. The experimentally induced rat lung silicosis model was successfully made by the instillation of silica intratracheally. This study was carried out based on the immunohistochemistry and the reverse transcription-polymerase chain reaction results, which increased the study's efficiency. Finally, all of the experiment (immunohistochemical staining experiments, RNA extraction, and RT-PCR) operation personnel and participants did not know the group status; therefore, the results of the study were objective.

- The study had also some limitations. First, due to sample size and experiment limitations, the number of grade two rats is fewer, so the present study did not analyze whether there is an association between the three factors and the silicosis grade. In addition, the study did not design the population-based method to discover the three factors' role in the development of silicosis simultaneously. Finally, based on current experimental design and analyses, it is impossible to verify whether the increase in TGF-B1, PDGF, and CTGF would also occur following exposure to other inflammatory stimuli other than silica

## 5. Conclusions

In vitro experiments showed that CTGF, TGF-*β*1, and PDGF increased in the lung of rats exposed to silica, and there was a positive feedback between these three cytokines. These results suggest that CTGF, TGF-*β*1, and PDGF may be cooperatively involved in the development process of pneumoconiosis induced by silica exposure. TGF-*β*/CTGF signaling pathway plays an important role in the process of pulmonary fibrosis, but its specific mechanism is still unclear. The findings may provide information for identification of silicosis-related biomarkers, which can be used in early diagnosis and health surveillance of silicosis.

## Figures and Tables

**Table 1:** Primer sequences, product length, and annealing temperature of RT-PCR.

Genes	Purpose product length (bp)	Annealing temperature (°C)	Upper primer (5′-3′)	Downstream primer (5′-3′)
ctgf	271	54.5	GTGTGCACTGCCAAAGATG	TCGGTAGGCAGCTAGGGC
tgf-*β*1	251	55	CCCAGCCTGCTTCTTGAGT	TCTCCCAAGGAAAGGTAGG
pdgf	289	57.3	AGAGCCTGCCG TAATCG	GGTCACTACTGTCTCACACTT
*β*-Actin	113	57	GCCCCTCTGAACCCTAA	GAGGCATACAGGGACAACA

**Table 2:** Comparison of the mRNA expression of CTGF, TGF-*β*1, and PDGF in rat lung ($\bar{X}\pm S$)^A^.

Cytokines	Experimental group	Control group	*P*
CTGF	0.823 ± 0.536	0.471 ± 0.238	0.012
TGF-*β*1	0.767 ± 0.510	0.401 ± 0.218	0.007
PDGF	1.179 ± 0.718	0.743 ± 0.435	0.027

^A^The data of this table were all in log scale.

**Table 3:** Correlations among the mRNA expression of CTGF, TGF-*β*1, and PDGF.

	Experimental group		Control group	
Between variables	Correlation coefficient	*P* ^B^	Correlation coefficient	*P* ^B^
CTGF∗TGF − *β*1	0.548	0.012	0.634	0.003
CTGF∗PDGF	0.489	0.029	0.507	0.023
TGF − *β*1∗PDGF	0.517	0.020	0.679	0.001

^B^Correlation is significant at the 0.05 level (2-tailed).

**Table 4:** Comparison of the positive area proteins of CTGF, TGF-*β*1, and PDGF in rat lung ($\bar{X}\pm S$)^C^.

Cytokines	Experimental group	Control group	*t*	*P*
CTGF	5.48 ± 0.23	4.68 ± 0.21	8.01	<0.0001
TGF-*β*1	5.84 ± 0.14	5.11 ± 0.22	8.43	<0.0001
PDGF	5.69 ± 0.13	4.78 ± 0.40	7.11	<0.0001

^C^The data of this table were all in log scale.

## Data Availability

The datasets used and analyzed during the current study are available from the corresponding author on reasonable request.
